# *Morchella importuna* Polysaccharides Alleviate Carbon Tetrachloride-Induced Hepatic Oxidative Injury in Mice

**DOI:** 10.3389/fphys.2021.669331

**Published:** 2021-08-03

**Authors:** Yingyin Xu, Liyuan Xie, Jie Tang, Xiaolan He, Zhiyuan Zhang, Ying Chen, Jie Zhou, Bingcheng Gan, Weihong Peng

**Affiliations:** ^1^National-Local Joint Engineering Laboratory of Edible and Medicinal Fungi, Agricultural Resources and Environment Institute, Sichuan Academy of Agricultural Science, Chengdu, China; ^2^Scientific Observing and Experimental Station of Agro-microbial Resource and Utilization in Southwest China, Ministry of Agriculture, Chengdu, China; ^3^Institute of Urban Agriculture, Chinese Academy of Agricultural Sciences, Chengdu, China

**Keywords:** *Morchella importuna*, liver, oxidative stress, inflammation, metabolomics, mice

## Abstract

This study aimed to investigate the effects of *Morchella importuna* polysaccharides (MIPs) on carbon tetrachloride (CCl_4_)-induced hepatic damage in mice. A total of 144 female mice were randomly assigned to four treatment groups, namely, control, CCl_4_, low-dose MIP (LMIP) group, and high-dose MIP (HMIP) group. After the 10-day experiment, serum and liver were sampled for biochemical and metabolomic analyses. The HMIPs markedly decreased the liver weight under CCl_4_ intoxication. Furthermore, the significantly elevated concentrations of five serum biochemical parameters, including alanine aminotransferase, aspartate aminotransferase, triglyceride, total cholesterol, and total bile acid under CCl_4_ treatment were subverted by MIP administration in a dose-dependent manner. Moreover, MIPs relieved the increased hepatic malonaldehyde and protein carbonyl content and the decreased superoxide dismutase and catalase contents caused by CCl_4_ intoxication. There was also a dose-dependent decrease in the CCl_4_-induced inflammatory indices, such as the levels of interleukin-1, interleukin-6, tumor necrosis factor-alpha, and myeloperoxidase, with MIP administration. Subsequent ultra-high performance liquid chromatography–tandem mass spectrometry-based serum metabolomics identified nine metabolites between the control and CCl_4_ groups and 10 metabolites between the HMIP and CCl_4_ groups, including some critical metabolites involved in flavonoid biosynthesis, amino acid metabolism, energy metabolism, and toxicant degradation. These novel findings indicate that MIPs may be of therapeutic value in alleviating the oxidative stress and inflammation caused by CCl_4_. Liquid chromatography-mass spectrometry-based metabolomics provides a valuable opportunity for identifying potential biomarkers and elucidating the protective mechanisms of medicinal mushrooms against hepatic oxidative injury.

## Introduction

The liver is considered a vital organ of the body with critical functions in protein synthesis, glucose homeostasis, detoxification, and nutrient utilization (Gao et al., 2008; Al-Seeni et al., 2016). The high levels of environmental toxins, alcohol, heavy metals, and metabolic dysfunction of the liver are among various insults that may lead to acute or chronic hepatic injury, followed by progressive fibrosis, cirrhosis, and even carcinoma (Sun et al., 2011; Nwokocha et al., 2012; Su et al., 2019; Chan et al., 2020).

Due to the implication of oxidative stress and inflammation in the etiology of liver injury, carbon tetrachloride (CCl_4_), a typical environmental toxicant, is widely used in experimental hepatopathy (Zou et al., 2016). CCl_4_-induced hepatotoxicity is attributed to reductive dehalogenation reactions catalyzed by the hepatic cytochrome P450, producing unstable trichloromethyl (CCl_3_•). Trichloromethyl peroxyl (Cl_3_COO•) radicals subsequently bind to proteins or lipids initiating lipid peroxidation and culminate in liver injury due to depletion of antioxidant enzymes (McGill and Jaeschke, 2013; Dong et al., 2015). During CCl_4_-induced oxidative damage, various inflammatory mediators, including interleukin-1beta (IL-1β), interleukin-6 (IL-6), and tumor necrosis factor-alpha (TNF-α), are released (Ashrafullah et al., 2018). Hepatic injury is further aggravated by the feedback mechanism.

Although some medicines have been developed to protect the liver, hepatoprotective drugs have potentially adverse side effects (Wang et al., 2019). Hence, finding natural plant extracts effective for the protection of liver injury is of intense interest.

Medicinal mushrooms have a long history of application in traditional oriental therapies, and fungal extracts are increasingly being employed to treat a wide range of diseases (Nakahara et al., 2020; Tung et al., 2020). *Morchella importuna* is a rare edible and medicinal mushroom species of true morels (the genus *Morchella*), highly appreciated for its commercial value, potent bioactivities, culinary qualities, and taste (He et al., 2019; Wang et al., 2020). Polysaccharide, an important active constituent of *M. importuna*, possesses various bioactivities, including antibacterial, antioxidant, anti-inflammatory, and antitumor properties, and a cardiovascular protective effect (Meng et al., 2018; Hou et al., 2020; Mu et al., 2020; Tang et al., 2020; Xue et al. 2019). This study has indicated that some plant polysaccharides also have hepatoprotective activity against drug- and toxicant-induced liver injuries. The polysaccharide of wild edible mushroom *Russula vinosa* protects the liver from CCl_4_-induced oxidative stress injury (Liu et al., 2014). Similarly, the antioxidant activities of *Cyclocarya paliurus* polysaccharide might be of therapeutic value in ameliorating the hepatic and renal oxidative stress caused by CCl_4_ (Wu et al., 2020b).

*Morchella importuna* polysaccharides (MIPs), with a molecular weight (Mw) of 28.5 kDa, are composed of *N*-acetylglucosamine, galactose, glucose, and mannose with molar ratios of 1.00:14.95:1.53:10.51 (Wen et al., 2019). These MIPs show strong antioxidant, immune-enhancing, hepatoprotective, and lipid-lowering activities. Therefore, it is attractive to further investigate their bioactivities, which provides feasible ideas and insights for the potential curative application of MIPs.

Accordingly, this study was performed to explore the effects of MIP administration on alleviating CCl_4_-induced oxidative stress and inflammation in mice. Subsequent metabolomic analysis was performed to uncover the potential pathways that were disturbed by CCl_4_ intoxication. These results provide partial theoretical evidence about the MIPs in preventing and treating oxidative injury in the liver.

## Materials and Methods

### Preliminary Characterization of MIPs

The Mw distribution of MIPs was determined by high-performance gel chromatography (HP-GPC) using a Waters 515 high-performance liquid chromatograph equipped with a laser detector, a differential refractive index, and a Shodex OHpak SB-806 gel chromatographic column (300 × 7.8 mm) set at 40°C ± 0.1°C. The mobile phase was 0.05 M NaH_2_PO_4_-NaH_2_PO_4_ buffer with 0.02% NaN_3_ (pH 6.7) and a flow rate of 0.5 ml/min. The volume of sample loaded was 500 μl. Polysaccharide standards with Mw values of 738, 5, 800, 1.22 × 10^4^, 2.37 × 10^4^, 4.8 × 10^4^, 1.0 × 10^5^, 1.86 × 10^5^, 3.8 × 10^5^, and 8.53 × 10^5^ g/mol were dissolved in the mobile phase. After filtration through a 0.45-μm filter, the samples and standards were analyzed under the abovementioned chromatographic conditions. Mw of the sample was calculated according to the plot of Mw vs. retention time of the standards.

The monosaccharide compositions of MIPs were analyzed by ion-exchange chromatography. After hydrolysis of the MIPs (5 mg) with 2 M of trifluoroacetic acid (TFA, 5 ml) at 120°C for 6 h, excess TFA was removed by co-distillation with methanol. The hydrolysate was redissolved in deionized water (1 ml) and passed through a 0.45-μm filter. High-performance anion-exchange chromatography-pulsed amperometric detection was carried out on a Dionex ICS-5000 system. Samples were eluted with a 91:9 (v/v) mixture of water and 200 mM NaOH.

### Analysis of MIPs by Fourier-Transform Infrared Spectroscopy

Spectroscopically, pure KBr was grounded in an agate mortar, then oven-dried at 110–150°C for 48 h, and stored in a dryer for use. Polysaccharide sample (5 mg) was mixed (1:100 w/w) with KBr powder and pressed into tablets. Scanning was carried out in the wavelength range of 4,000–400 cm^−1^ at a resolution of 4 cm^−1^.

### Animals

Female C57BL/6 mice (17 ± 0.28 g) were purchased from Dossy Experimental Animals Co., Ltd. (Chengdu, China). Before the experiment, all mice were conditioned for 1 week under a 12-h light/12-h dark cycle, at 22 ± 3°C, and 50–70% relative humidity, with free access to water and food. The experimental protocol was approved by the Ethics Review Committee for Animal Experimentation of Sichuan Academy of Agricultural Sciences. All procedures followed the Guide for the Care and Use of Laboratory Animals (National Institutes of Health).

### Experimental Design

In a 10-day experiment, 144 mice were randomly assigned to four treatments with six replications per treatment (six mice per replication) as follows: control (*n* = 36), orally administered saline once a day; CCl_4_ (*n* = 36), orally administered saline once a day; LMIPs (*n* = 36), orally administered saline and MIPs at 100 mg/kg body weight (BW) once a day; HMIPs (*n* = 36), orally administered saline and MIPs (200 mg/kg BW) once a day. At 2 h after the last administration, all mice (except for the control group) were intraperitoneally injected with CCl_4_ (0.2% CCl_4_/olive oil mixture) at 5 ml/kg BW. The mice in the control group were intraperitoneally injected only with the same volume of olive oil.

### Sample Collection

All mice fasted for 16 h after treatment. One mouse with the average BW within each replication was anesthetized by the intraperitoneal injection of 500 μl of pentobarbital sodium (8 mg/kg BW). Venous blood (1 ml) was collected and centrifuged at 3,000 *g* and 4°C for 10 min to obtain serum. The liver tissues were harvested for biochemical or histopathological analysis.

### Biochemical Analysis

Serum contents of alanine aminotransferase (ALT), aspartate aminotransferase (AST), triglyceride (TG), total cholesterol (TC), and total bile acid (TBA) were determined using spectrophotometric kits according to the instructions of the manufacturer (Nanjing Jiancheng Bioengineering Institute, Nanjing, China).

Hepatic tissues were homogenized in saline and centrifuged at 2,500 *g* for 10 min to collect the supernatant for the quantitation of malonaldehyde (MDA), protein carbonyl (PC), superoxide dismutase (SOD), catalase (CAT), IL-1, IL-6, TNF-α, and myeloperoxidase (MPO) by spectrophotometric methods using commercially available kits (Nanjing Jiancheng Bioengineering Institute, Nanjing, China).

### Histopathological Analysis

The liver tissue was fixed in a 4% paraformaldehyde solution for 48 h, then dehydrated, and embedded in the wax block. It was cut into 5-μM thick sheets and stained with H&E. Histopathological sections were viewed under a light microscope at optical magnifications of 100 and 400 ×.

### Serum Metabolomic Analysis

#### Sample Pre-treatment

Serum (100 μl) and pre-chilled methanol (400 μl; Thermo Fisher, Massachusetts, USA) were mixed thoroughly with a vortex. The samples were incubated on ice for 5 min, and then centrifuged at 15,000 rpm and 4°C for 5 min. Some supernatants were diluted with liquid chromatography-mass spectrometry (LC-MS)-grade water (Thermo Fisher) to obtain a final concentration of 60% methanol. These samples were subsequently transferred to a fresh Eppendorf tube with a 0.22-μm filter and then centrifuged at 15,000 *g* and 4°C for 10 min. Finally, the filtrate was injected into the liquid chromatography-tandem mass spectrometry (LC-MS/MS) system.

#### Liquid Chromatography-Tandem Mass Spectrometry Analysis

The LC-MS/MS analysis was performed using a Vanquish UHPLC system (Thermo Fisher) coupled with an Orbitrap Q Exactive HF-X mass spectrometer (Thermo Fisher). Samples were injected onto a Hypersil Gold column (100 × 2.1 mm, 1.9 μm) and eluted by a 16-min linear gradient at a flow rate of 0.2 ml/min. The eluents for the positive polarity mode were eluent A (0.1% formic acid in water) and eluent B (methanol). The eluents for the negative polarity mode were eluent A (5 mM ammonium acetate, pH 9.0) and eluent B (methanol). The solvent gradient was set as follows: 2% B, 1.5 min; 2–100% B, 12.0 min; 100% B, 14.0 min; 100–2% B, 14.1 min; and 2% B, 16.0 min. The Q Exactive HF-X mass spectrometer was operated in a positive/negative polarity mode with a spray voltage of 3.2 kV, a capillary temperature of 320°C, a sheath gas flow rate of 35 arb, and an aux gas flow rate of 10 arb.

#### Data Analysis

The raw data files generated by ultra-high performance liquid chromatography-tandem mass spectrometry (UHPLC–MS/MS) were processed using the Compound Discoverer 3.0 (CD3.0, Thermo Fisher) to perform peak alignment, peak picking, and quantitation for each metabolite.

These metabolites were annotated using the Kyoto Encyclopedia of Genes and Genomes (KEGG) database (http://www.genome.jp/kegg/), Human Metabolome Database (http://www.hmdb.ca/), and LIPID MAPS database (http://www.lipidmaps.org/). Principal component analysis (PCA) and partial least square-discriminant analysis (PLS-DA) were performed using metaX (a flexible and comprehensive software for processing metabolomic data). We applied univariate analysis (*t*-test) to calculate the statistical significance (*P*-value). The metabolites with variable importance in the projection (VIP) > 1, *P* < 0.05, and fold change (FC) ≥2 or ≤0.5 were considered to be differential metabolites. Volcano plots filtered out the metabolites of interest that met the VIP, log_2_(FC), and –log_10_ (*P*-value) criteria.

For hierarchal clustering and heat map generation, the data were normalized using *z*-scores of the intensity areas of differential metabolites and plotted using the Pheatmap package in R software. The correlation between differential metabolites was analyzed using the cor() function in R (Pearson's method). Statistically significant (*P* < 0.05) correlations between differential metabolites were calculated using cor.mtest() function in R. Correlation plots were plotted using the corrplot package in R. The functions of these metabolites and metabolic pathways were studied using the KEGG database. The metabolic pathway enrichment analysis of differential metabolites was performed when the ratio was satisfied by *x/n* > *y/N*, and the metabolic pathway was considered significantly enriched when *P* < 0.05.

### Statistical Analysis

All data were assessed by ANOVA and Duncan's multiple range test using SPSS 23.0 analysis software at *P* < 0.05 (IBM Corp., New York, USA). One mouse was selected from each replication as an experimental unit. Data were expressed as mean ± SD.

## Results

### Determination of Mw and Monosaccharide Composition of MIPs

From the HP-GPC analysis, the Mw of MIPs was 35.54 kDa, the number-average molecular weight (Mn) was 34.77 kDa, and the *D*-value (Mw/Mn) was 1.002. These results indicated that the MIPs were relatively pure because the polydispersity ratio was close to 1, and the Mw distribution was narrow. Further structural analysis showed that the monosaccharide molar ratios of the MIPs were 47.1:30.4:12.5:10.0 (Table 1).

**Table 1:** Molecular weights and compositions of MIPs.

**Sample**	**MIPs**
Mw (kDa)	35.54
Mn (kDa)	34.77
D-value	1.002
Mw distribution%	
28 700.0–34 000.0 g/mol	47.1
34 000.0–40 000.0 g/mol	30.4
40 000.0–43 000.0 g/mol	12.5
43 000.0–47 738.0 g/mol	10.0

*MIPs, Morchella importuna polysaccharides; Mw, weight-average molecular weight; Mn, number-average molecular weight; D = Mw/Mn*.

### Infrared Spectrum Analysis of MIPs

**Figure 1:**
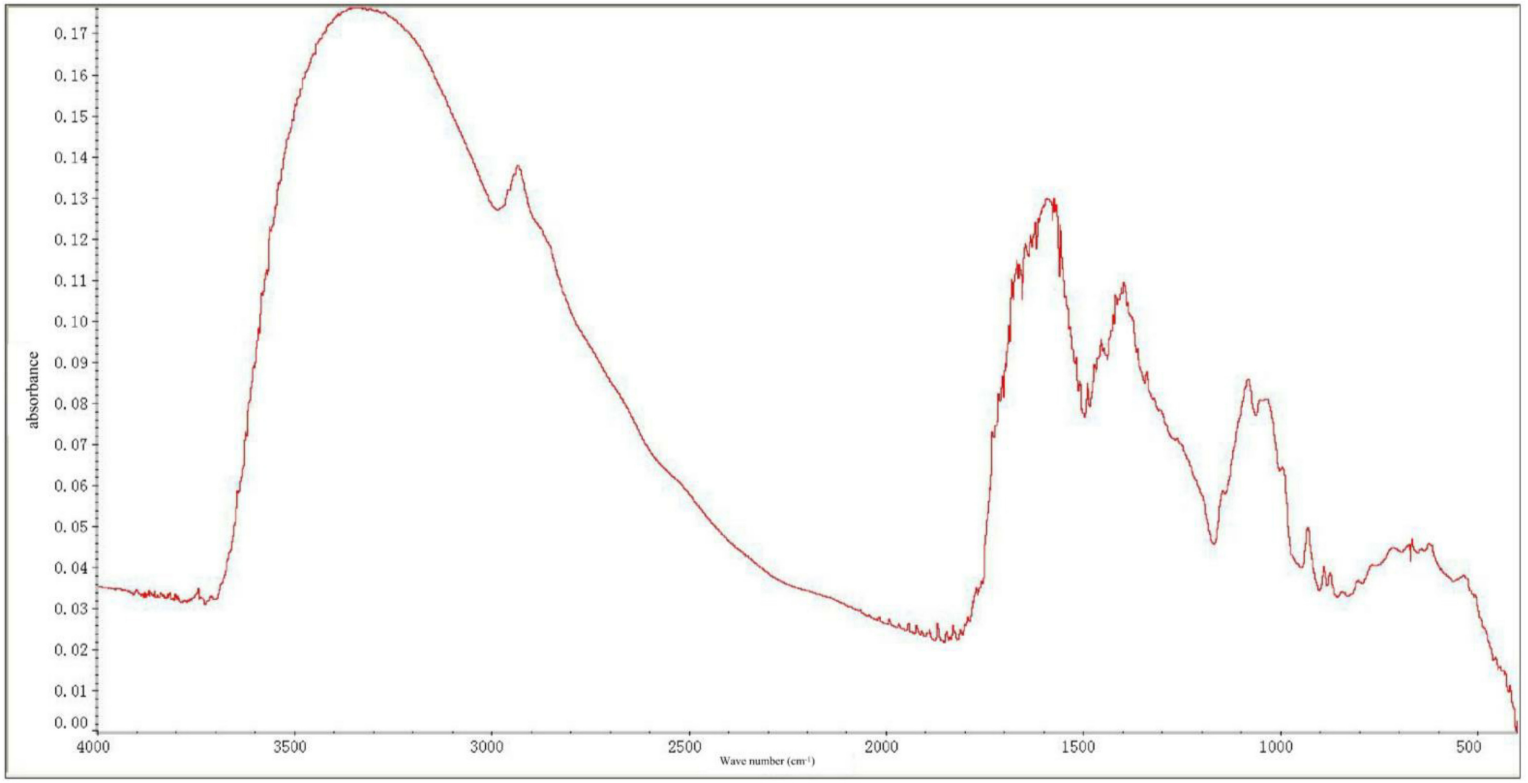
Fourier-transform infrared spectra of *Morchella importuna* polysaccharides (MIPs).

Infrared spectroscopy is a powerful technique for analyzing the structure of polysaccharides. From the infrared spectrum analysis (Figure 1), the absorption peaks at 3,100–3,000 cm^−1^ (C–H stretching vibration), 1,675–1,640 cm^−1^ (C=C stretching vibration), and 1,000–675 cm^−1^ (out-of-plane bending vibration of olefin C–H) were ascribed to the double bonds in the MIPs. The strong absorption peaks at 3,100–3,000 cm^−1^ (C–H stretching vibration on the aromatic ring), unequal absorption (C=C) at 1,600, 1,580, 1,500, and 1,450 cm^−1^, and the absorption peaks at 1,000–675 cm^−1^ indicated that aromatic rings exist in the MIPs. The absorption peaks at 3,650–3,600 cm^−1^, 1,300–1,000, and 769–659 characterized the existence of alcoholic hydroxyl and phenol groups. The absorption peaks at 1,750–1,700 cm^−1^ (C=O) and 2,820 and 2,720 (aldehyde group C–H) depicted the existence of the aldehyde groups. There was also a strong absorption peak at 1,715 cm^−1^ (keto group). Strong absorption at 3,300–2,500 cm^−1^, C=O stretching absorption at 1,720–1,706, C–O stretching absorption at 1,320–1,210 cm^−1^, and absorption at 920 cm^−1^ highlighted the existence of carboxyl groups.

### Effects of MIP Administration on the Body and Liver Weights

**Figure 2:**
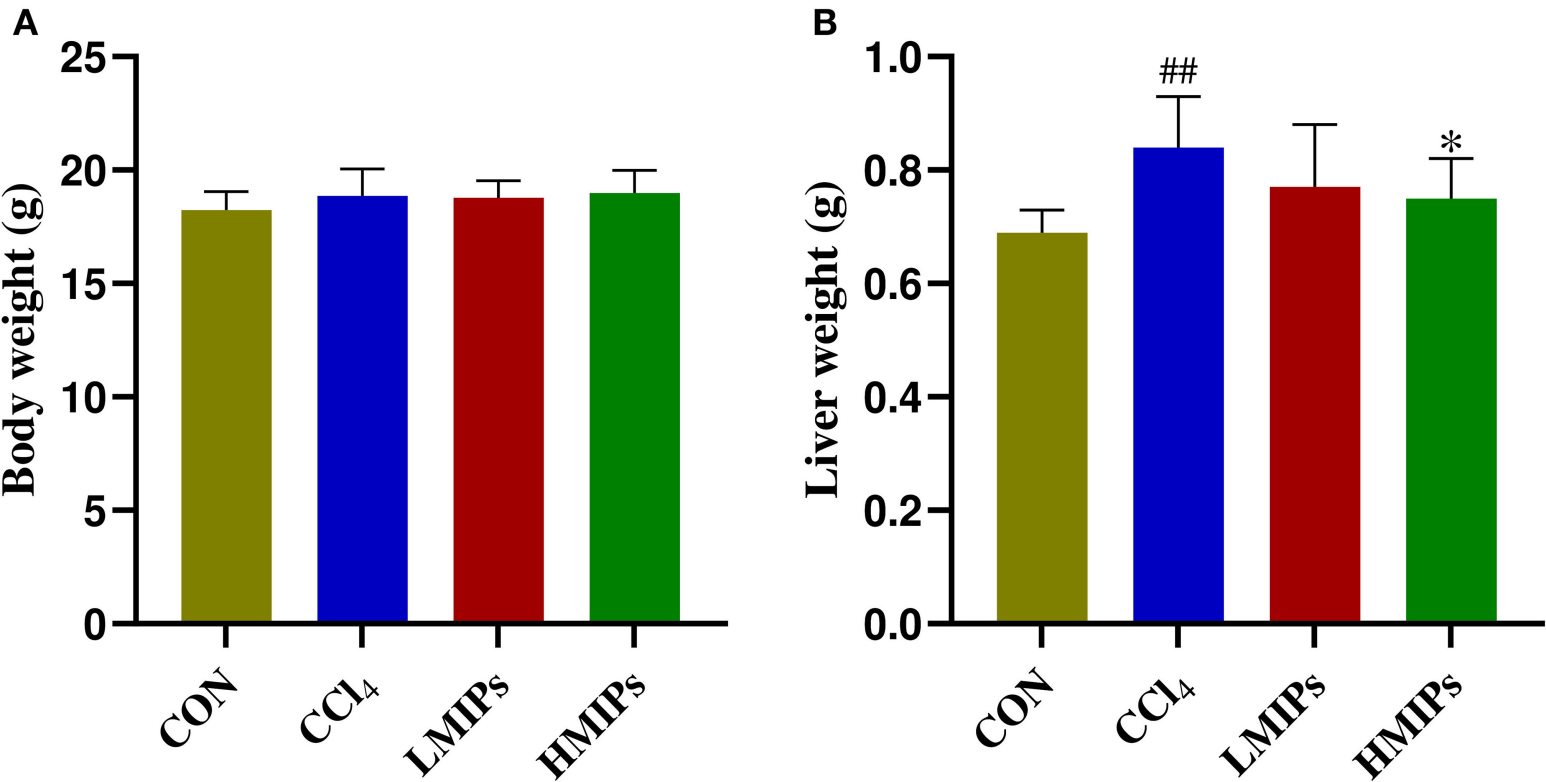
Effects of MIP infusion on the body **(A)** and liver weight **(B)**. The values are reported as the mean ± SD of six mice per group: (##) *P* < 0.01 compared with the control group and (*) *P* < 0.05 compared with the carbon tetrachloride (CCl_4_)-treated group.

The body and liver weights were determined to assess the effect of MIP administration following hepatic injury induced by CCl_4_. As shown in Figure 2A, there were no obvious changes in BW among the four groups. However, a significant (*P* < 0.01) increase in liver weight was observed in the CCl_4_-treated mice compared with the control group (Figure 2B). HMIP administration significantly decreased liver inflammation and, in turn, liver weight (*P* < 0.05).

### Effect of MIP Administration on Serum Biochemical Parameters

**Figure 3:**
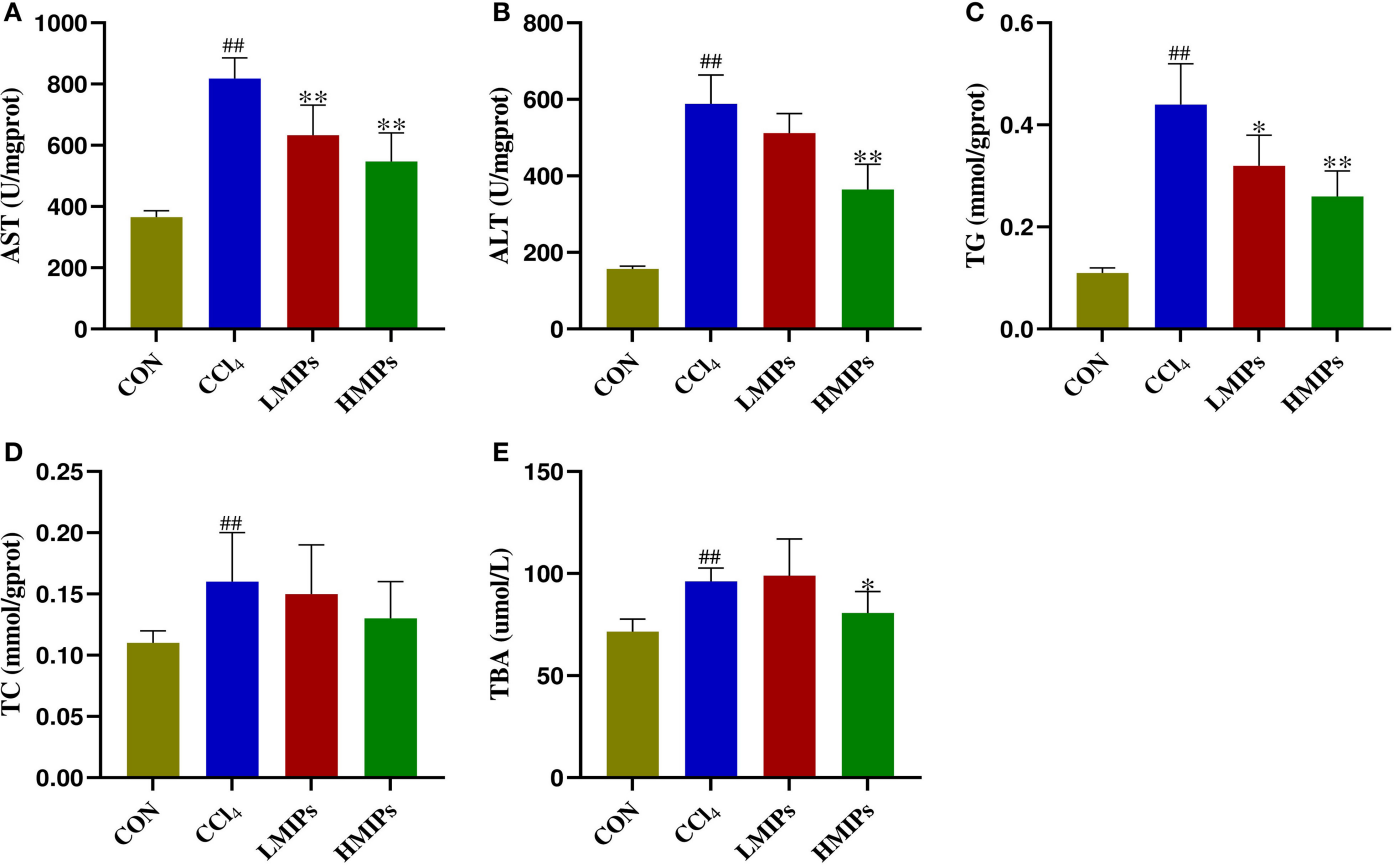
Effect of MIP infusion on serum biochemical parameters. The content of serum aspartate aminotransferase **(A)**, alanine aminotransferase **(B)**, triglyceride **(C)**, total cholesterol **(D)**, and total bile acid **(E)** was determined by ELISA kits. The values are reported as the mean ± SD of six mice per group: (##) *P* < 0.01 compared with the control group and (**) *P* < 0.01 and (*) *P* < 0.05 compared with the CCl_4_-treated group.

To clarify the effects of MIP administration on the CCl_4_-induced liver injury markers, we measured the biochemical indices of serum samples. The results (Figure 3) showed that the levels of serum AST, ALT, TG, TC, and TBA were elevated by CCl_4_ intoxication. Conversely, pre-treatment with MIPs dose-dependently alleviated the increase in AST, ALT, TG, and TBA, with a more significant (*P* < 0.01) result at a higher dose (200 mg/kg BW).

### Effect of MIP Administration on Hepatic Antioxidant Activity

**Figure 4:**
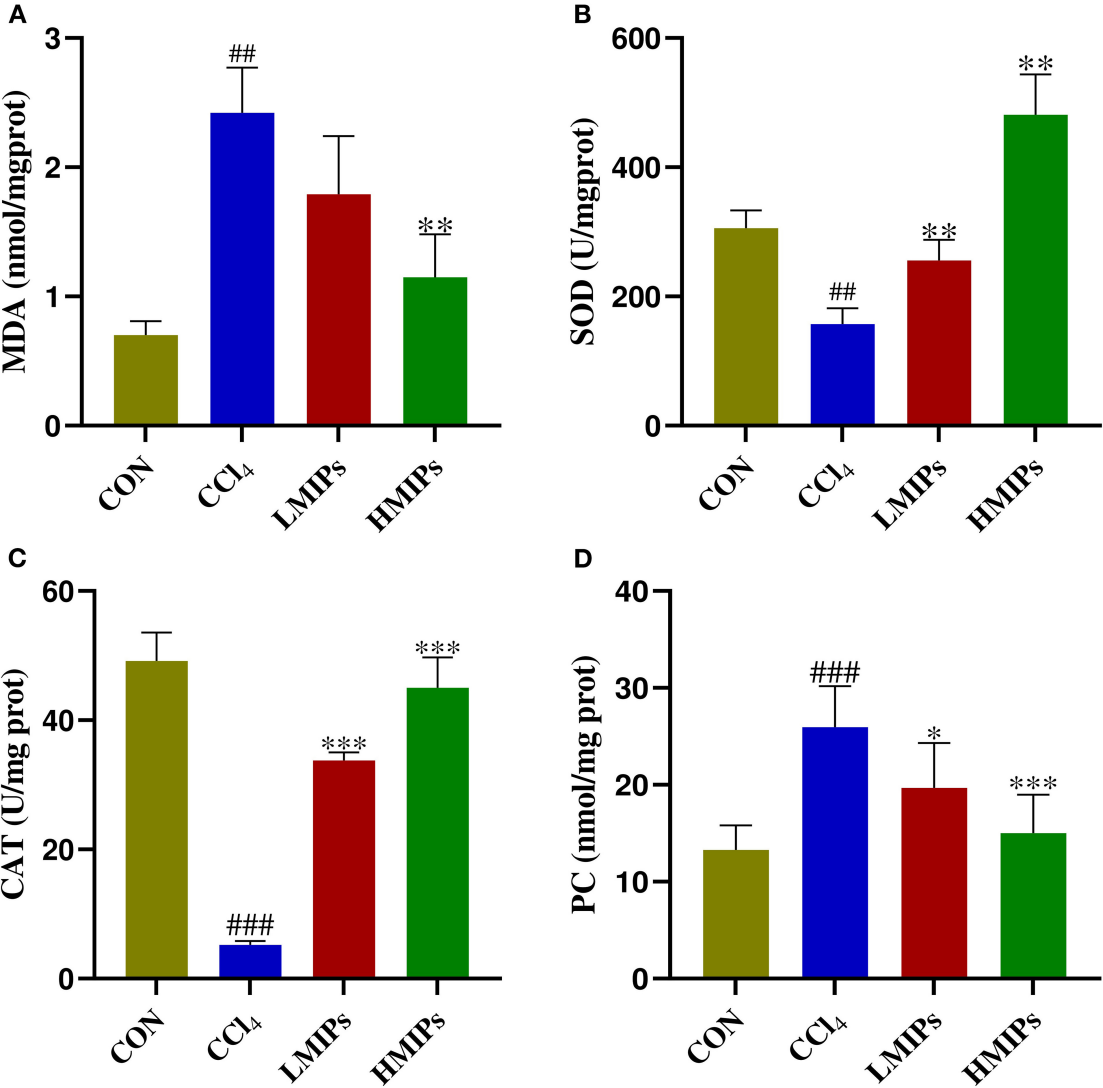
Effect of MIP infusion on hepatic antioxidant activity. The content of hepatic malonaldehyde **(A)**, superoxide dismutase **(B)**, catalase **(C)**, and protein carbonyl **(D)** was determined by ELISA kits. The values are reported as the mean ± SD of six mice per group: (#) *P* < 0.05 compared with the control group, (##)*P* < 0.01. (*)*P* < 0.05 compared with the CCl4-treated group, (**) *P* < 0.01 and (***) *P* < 0.001.

Biomarkers of membrane lipid peroxidation and protein oxidative injury (MDA and PC) were examined. As illustrated in Figures 4A,D, CCl_4_ directly induced a significant increase in hepatic MDA and PC contents (*P* < 0.01), which were restored by MIP administration in a dose-dependent manner (*P* < 0.01). The activities of SOD and CAT in the experimental animals are shown in Figures 4B,C. Compared with the control group, the acute administration of CCl_4_ to mice induced characteristic hepatotoxicity that affected hepatic antioxidant parameters, as indicated by a significant decrease in SOD and CAT levels (*P* < 0.01). These CCl_4_-induced declines were markedly attenuated by pre-treatment with LMIPs and HMIPs (*P* < 0.01).

### Effect of MIP Administration on Hepatic Inflammation

**Figure 5:**
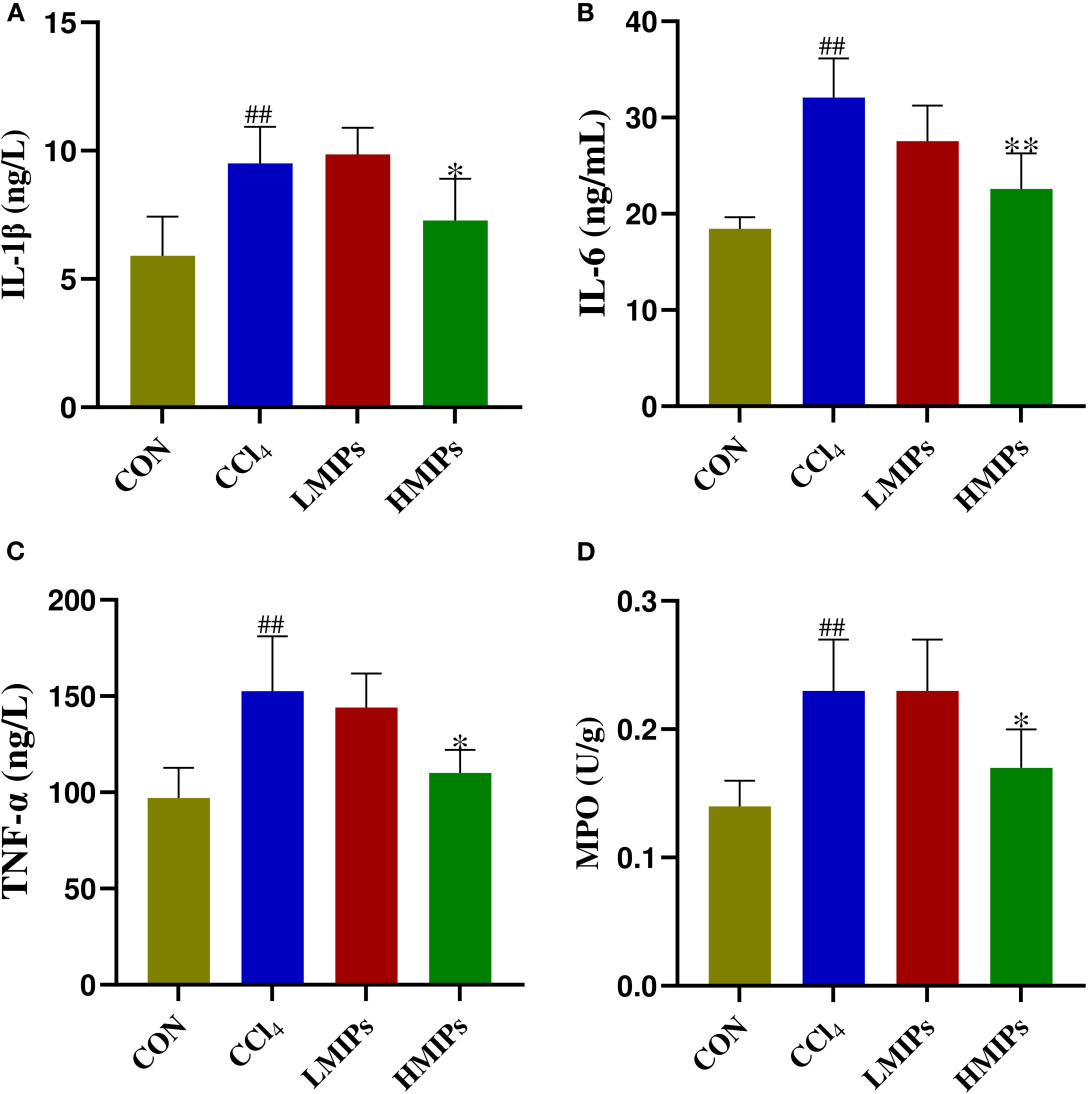
Effect of MIP infusion on hepatic inflammation. The content of hepatic interleukin-1beta **(A)**, interleukin-6 **(B)**, tumor necrosis factor-alpha **(C)**, and myeloperoxidase **(D)** was determined by ELISA kits. The values are reported as the mean ± SD of six mice per group: (##) *P* < 0.01 compared with the control group and (**) *P* < 0.01 and (*) *P* < 0.05 compared with the CCl_4_-treated group.

We also assessed the effect of the MIP administration on the production of proinflammatory mediators (i.e., IL-1β, IL-6, and TNF-α) after CCl_4_ intoxication. In this study, IL-1β (Figure 5A), IL-6 (Figure 5B), and TNF-α (Figure 5C) levels were markedly increased in the CCl_4_ group. MIP administration attenuated the production of these cytokines in a dose-dependent manner. Additionally, MPO activity, the marker of neutrophilic infiltration, was assayed to evaluate the hepatic injury. CCl_4_ intoxication led to an evident accumulation of MPO in the liver (Figure 5D). In contrast, MIP administration resulted in a dose-dependent diminution of MPO, with a more significant effect at a higher dose (200 mg/kg).

### Histopathological Detection

**Figure 6:**
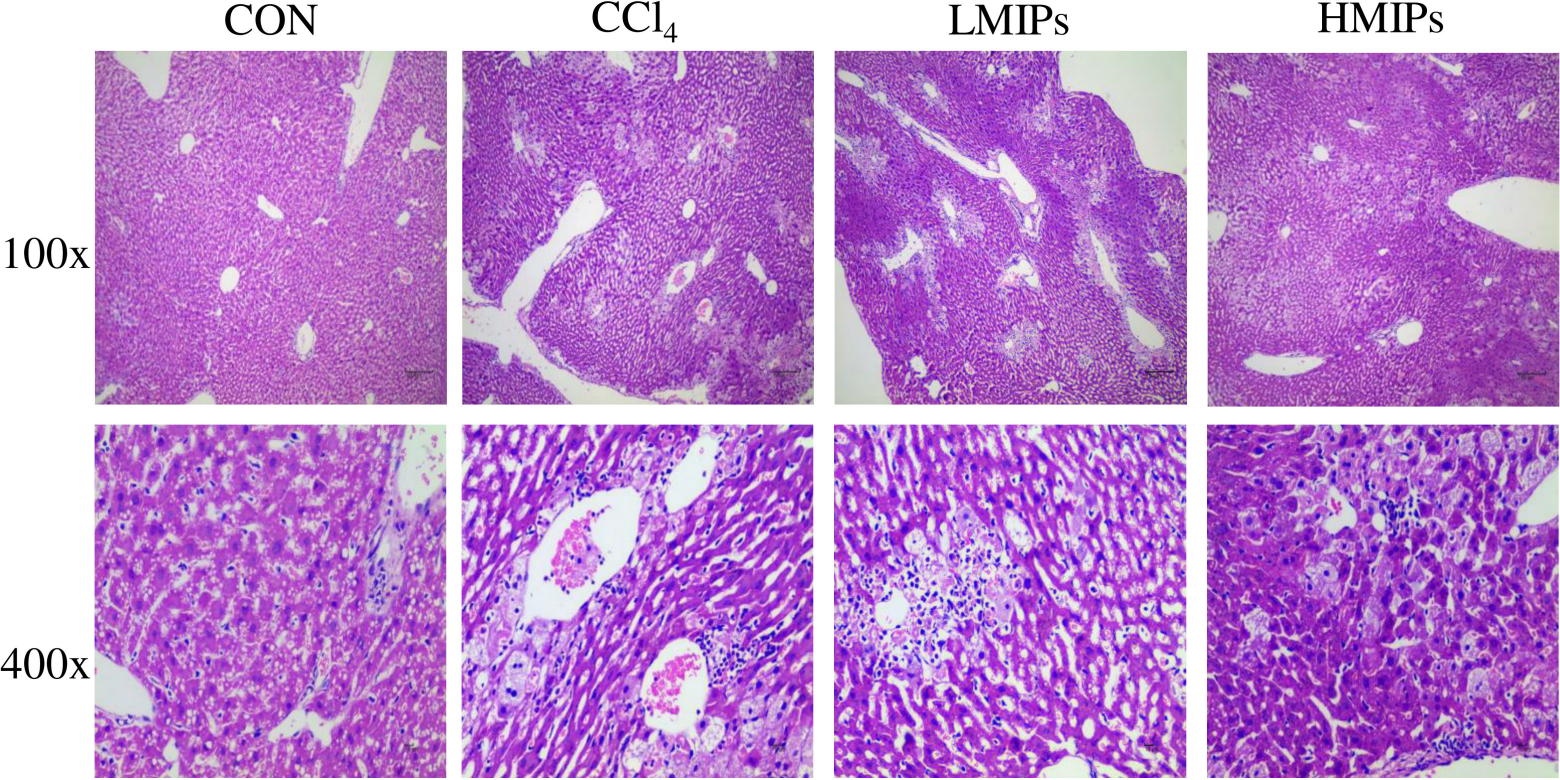
Effect of MIPs on the liver as disclosed by morphological analysis (H and E staining, magnifications of 100× and 400×).

In the control group, structurally ordered tissues and hepatocytes with prominent nuclei and uniform cytoplasm were observed under the optical microscope. Acute administration of CCl_4_ induced substantial liver damage, as evidenced by massive hepatocyte necrosis with inflammatory cell infiltration and hepatocyte ballooning degeneration. However, the abnormal status was ameliorated by the HMIP administration. Hepatic architecture distortion was relieved, as indicated by decreased cellular necrosis, inflammatory infiltration, and ballooning degeneration (Figure 6).

### Metabolic Profiling Comparison Among the Control, CCl_4_, and HMIP Groups

**Figure 7:**
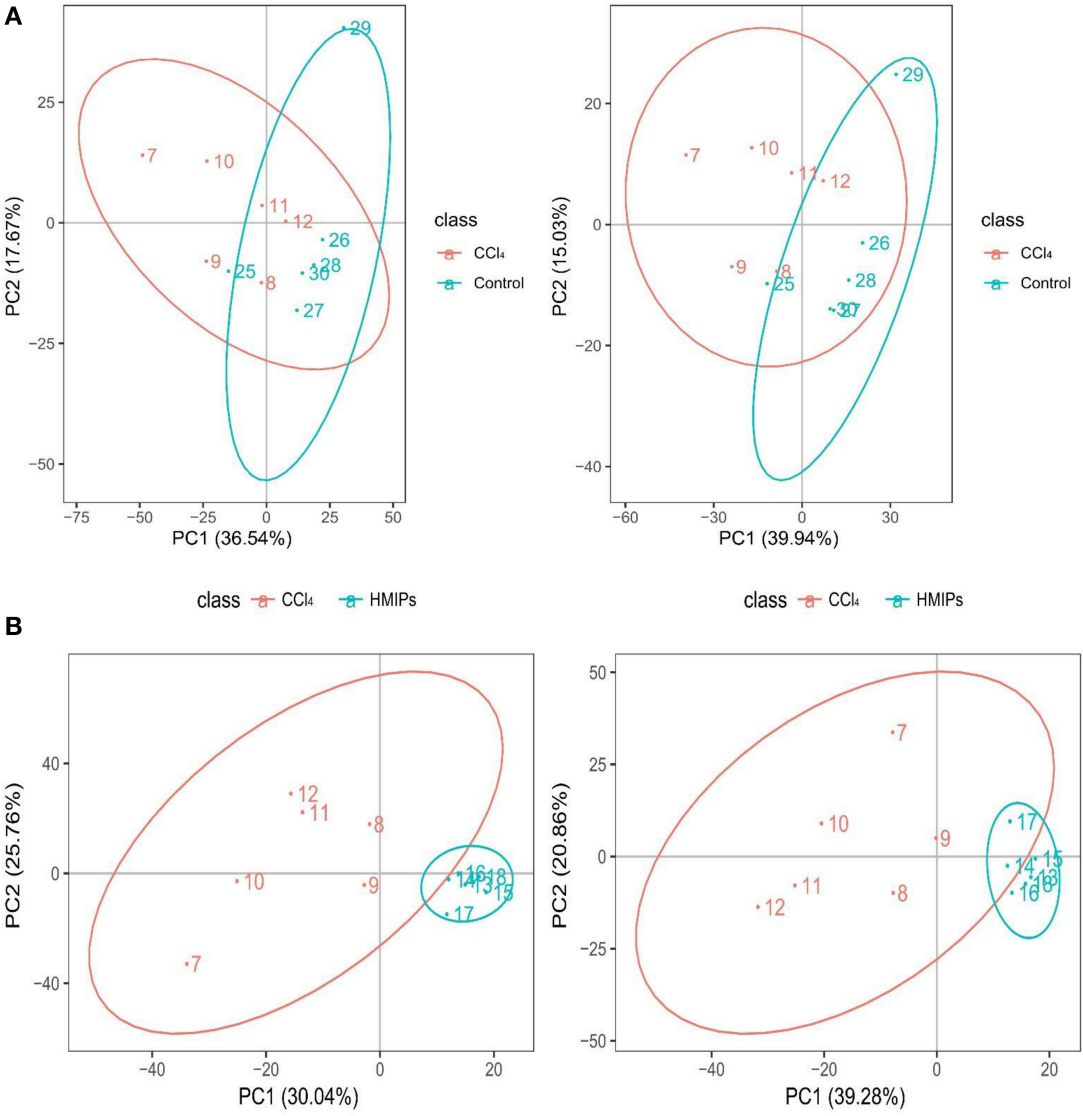
Principal component analysis (PCA) score plots of the serum samples. **(A)** PCA score plots of the control and CCl_4_ groups under the positive and negative ion models. **(B)** PCA score plots of the HMIP and CCl_4_ groups under the positive and negative ion models.

**Figure 8:**
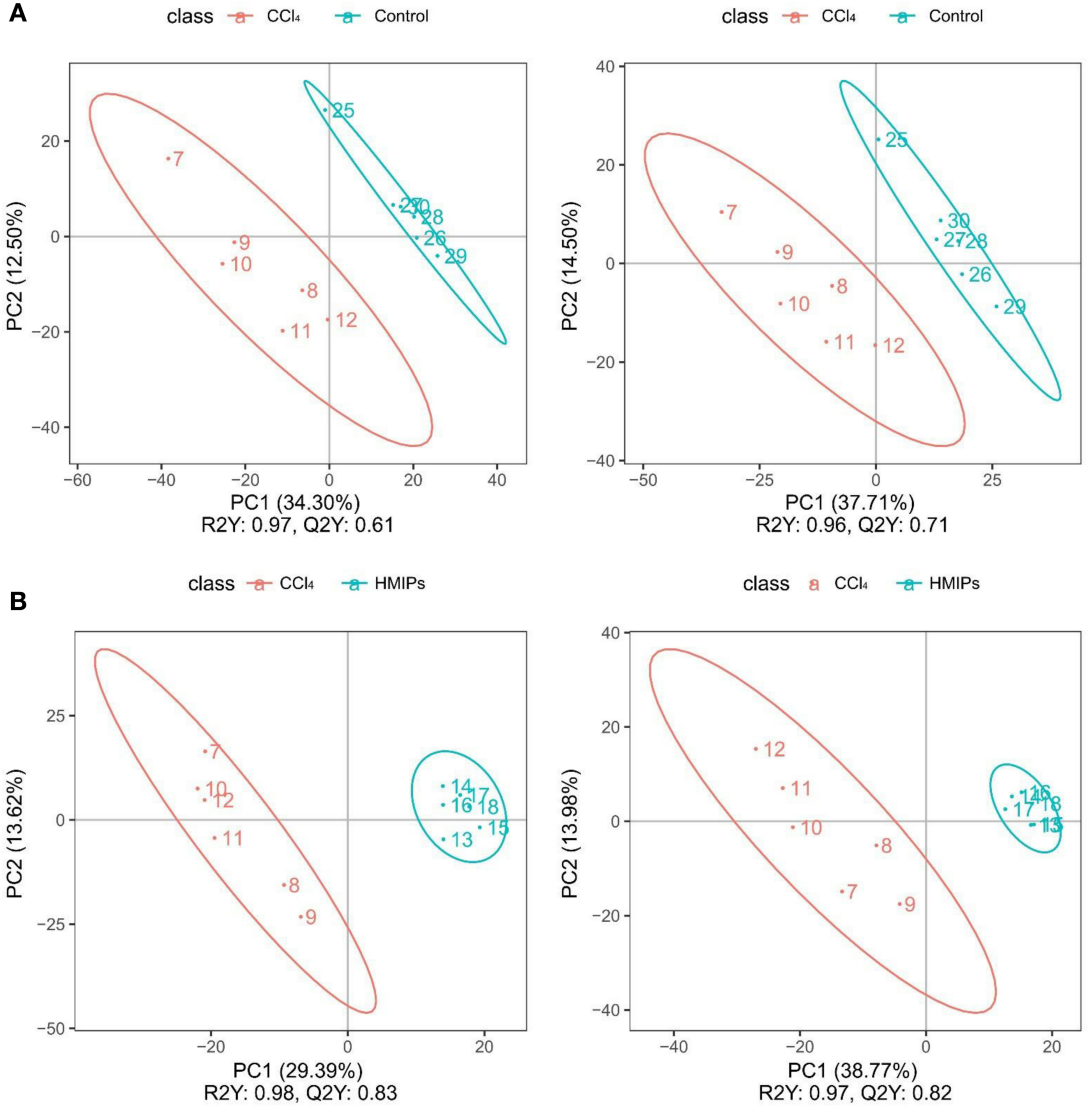
Partial least squares-discriminant analysis (PLS-DA) score plots of the serum samples. **(A)** PLS-DA score plots of the control and CCl_4_ groups under the positive and negative ion models. **(B)** PLS-DA score plots of the HMIP and CCl_4_ groups under the positive and negative ion models.

The PCA and PLS-DA were used to study the differences in serum metabolite profiles among the control, CCl_4_, and HMIPs groups. The PCA score plots of the serum-based LC-MS data showed an obvious distinction among the three groups (Figure 7). The PLS-DA score plot for the three groups revealed visible separations of the serum metabolites (Figure 8), indicating that HMIP administration could partly regulate the metabolic disorders caused by CCl_4_.

### Effect of HMIP Administration on the Metabolic Profiles

**Figure 9:**
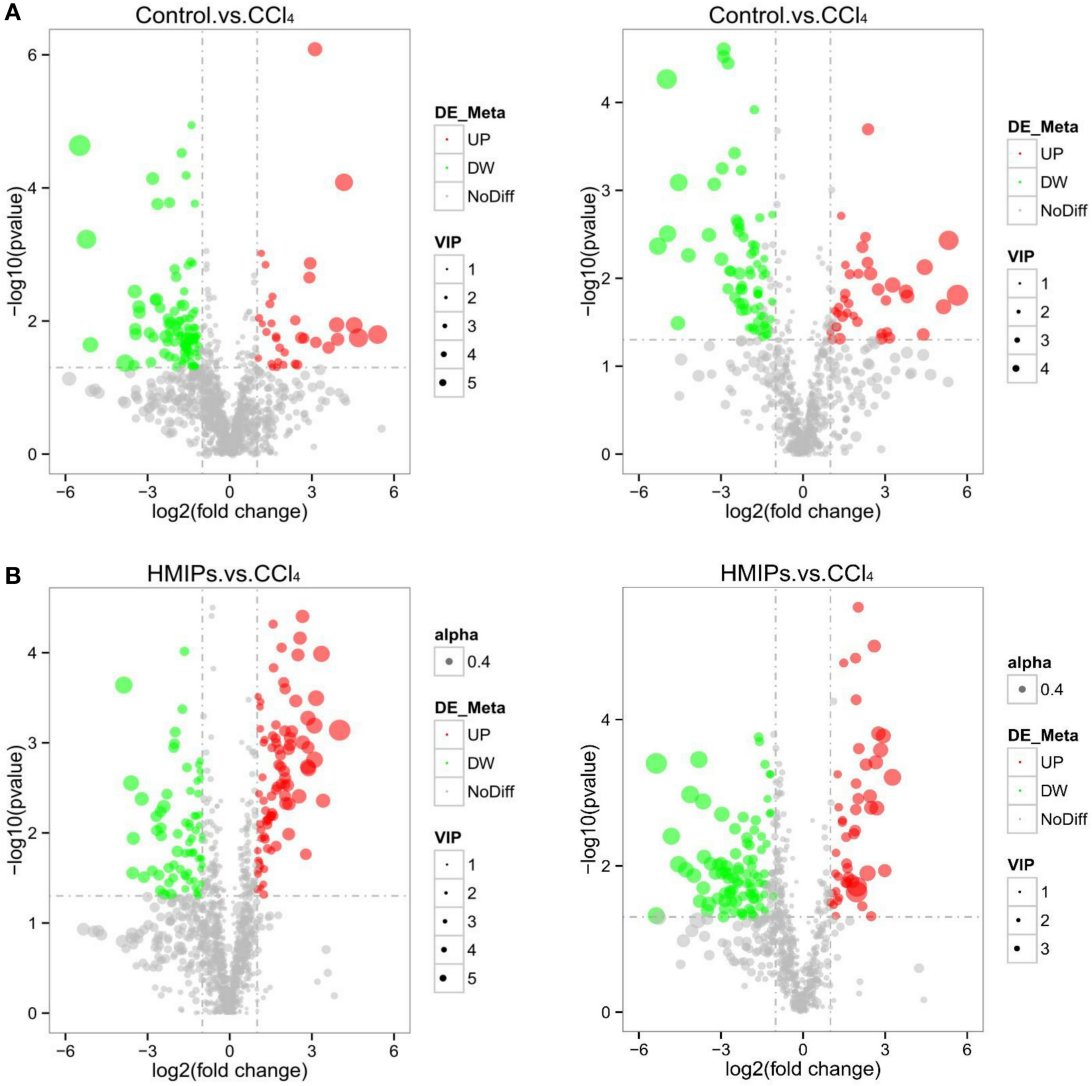
Volcano plot of the serum samples. **(A)** Volcano plot of the control and CCl_4_ groups under the positive and negative ion models. **(B)** Volcano plot of the HMIP and CCl_4_ groups under the positive and negative ion models. Significantly increased (decreased) variables are represented in the red (green) circle.

**Figure 10:**
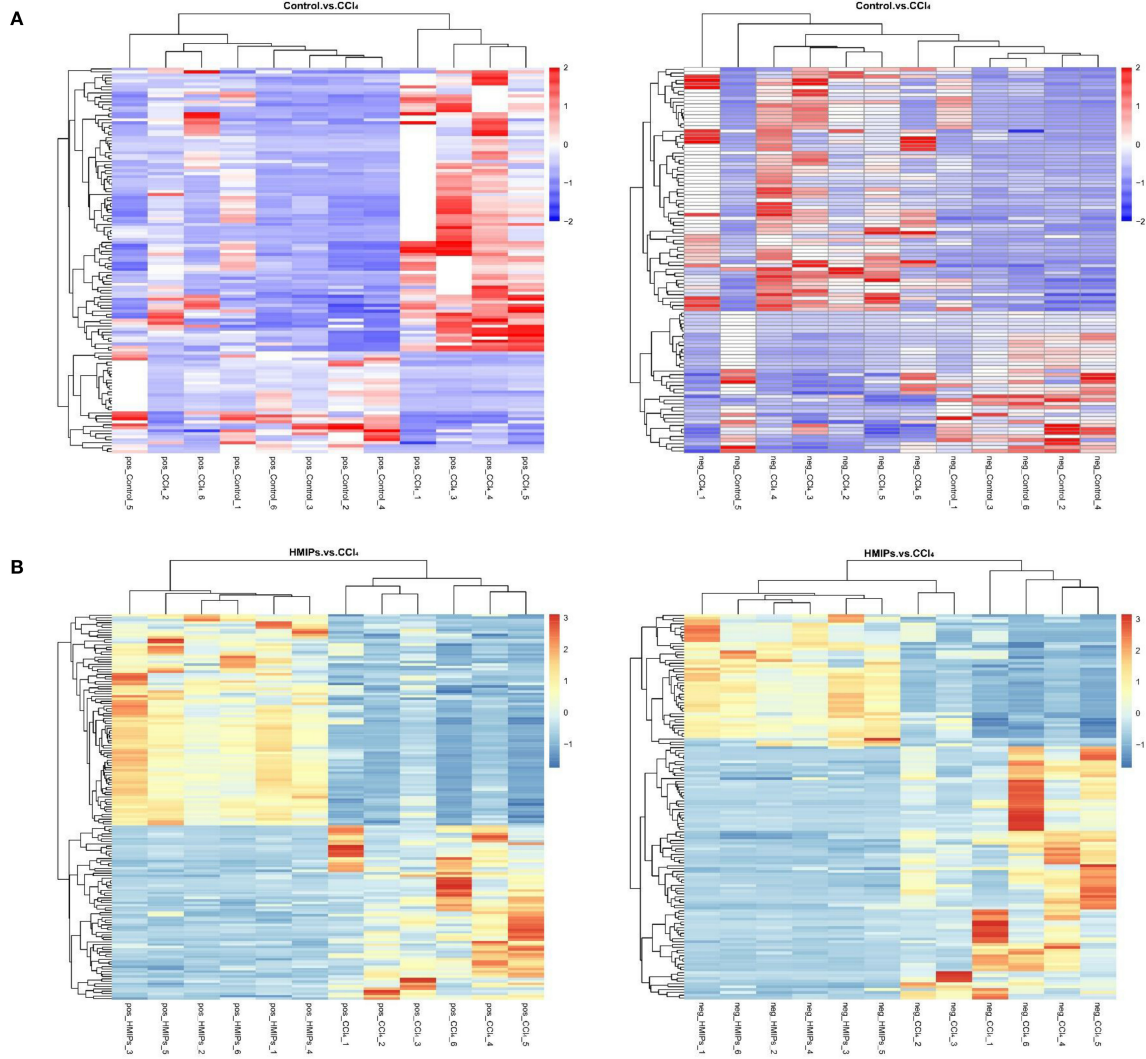
Heat map of hierarchical clustering analysis (HCA) for differential metabolites. **(A)** Heat map of the control and CCl_4_ groups under the positive and negative ion models. **(B)** Heat map of the HMIP and CCl_4_ groups under the positive and negative ion models. Colors represent signal intensities from 0% (blue) to 100% (red).

In the PLS-DA score plot, variables that significantly contributed to the clustering and discrimination were identified according to a VIP threshold of ≥1.0. When the *P*-value was lower than 0.05, the compounds were screened as candidate differential metabolites for one-way ANOVA analysis. The results were visualized in the form of a volcano plot (Figure 9). There were 9 differential metabolites between the control and CCl_4_ groups and 10 differential metabolites between HMIP and CCl_4_ groups (Tables 2, 3). For hierarchal clustering and heat map generation, the data were calculated and plotted in R software. It showed that the differential metabolite profiles differed among the three groups (Figure 10). These results indicate that HMIPs effectively prevent CCl_4_-induced liver injury via mitigating the altered serum metabolite profiles and regulating metabolic abnormalities.

**Table 2:** Summary of the candidate biomarkers between control and CCl_4_ groups.

**Metabolite**	**Formula**	**Mass**	**RT (min)**	**Ratio**	***P*-value**	**Pathways**
Isoliquiritigenin	C_15_H_12_O_4_	256.07	8.75	8.85	0.021	Flavonoid biosynthesis
Prunin	C_21_H_22_O_10_	434.12	9.00	3.96	0.031	Flavonoid biosynthesis
Calcitriol	C_27_H_44_O_3_	416.33	14.00	2.48	0.001	Steroid biosynthesis
γ-Aminobutyric acid	C_4_H_9_NO_2_	103.06	1.10	0.46	0.002	Alanine, aspartate and glutamate metabolism
Deoxycholic acid	C_24_H_40_O_4_	392.29	11.59	0.04	0.03	Secondary bile acid biosynthesis
6-Hydroxynicotinic acid	C_6_H_5_NO_3_	139.03	1.22	0.33	<0.001	Nicotinate and nicotinamide metabolism
Trigonelline	C_7_H_7_NO_2_	137.05	1.09	5.26	0.001	Nicotinate and nicotinamide metabolism
Hydroquinone	C_6_H_6_O_2_	110.04	5.90	4.07	0.009	Benzoate and aminobenzoate degradation
Catechol	C_6_H_6_O_2_	11.04	2.75	6.71	0.014	Benzoate and aminobenzoate degradation

*RT, retention time*.

**Table 3:** Summary of the candidate biomarkers between HMIP and CCl_4_ groups.

**Metabolites**	**Formula**	**Mass**	**RT (min)**	**Ratio**	***P*-value**	**Pathways**
Prunin	C_21_H_22_O_10_	434.12	9.00	0.15	0.013	Flavonoid biosynthesis
Xanthurenic acid	C_10_H_7_NO_4_	205.04	7.47	0.38	0.012	Tryptophan metabolism
Kynurenic acid	C_10_H_7_NO_3_	189.04	7.58	0.25	<0.01	Tryptophan metabolism
Serotonin	C_10_H_12_N_2_O	176.10	1.10	0.33	0.030	Tryptophan metabolism
Hippuric acid	C_9_H_9_NO_3_	179.06	6.95	0.18	0.011	Phenylalanine metabolism
Succinic acid	C_4_H_6_O_4_	118.03	1.57	0.09	0.028	Phenylalanine metabolism
Phenylacetylglycine	C_10_H_11_NO_3_	193.07	7.54	0.50	0.012	Phenylalanine metabolism
Trigonelline	C_7_H_7_NO_2_	137.05	1.09	0.42	0.040	Nicotinate and nicotinamide metabolism
2-Naphthalenesulfonic acid	C_10_H_8_O_3_S	208.02	7.87	0.35	0.006	Naphthalene degradation
Catechol	C_6_H_6_O_2_	110.04	2.75	0.22	0.05	Benzoate and aminobenzoate degradation

*HMIPs, high concentration of Morchella importuna polysaccharides; RT, retention time*.

### Enrichment Analysis of Metabolic Pathway

**Figure 11:**
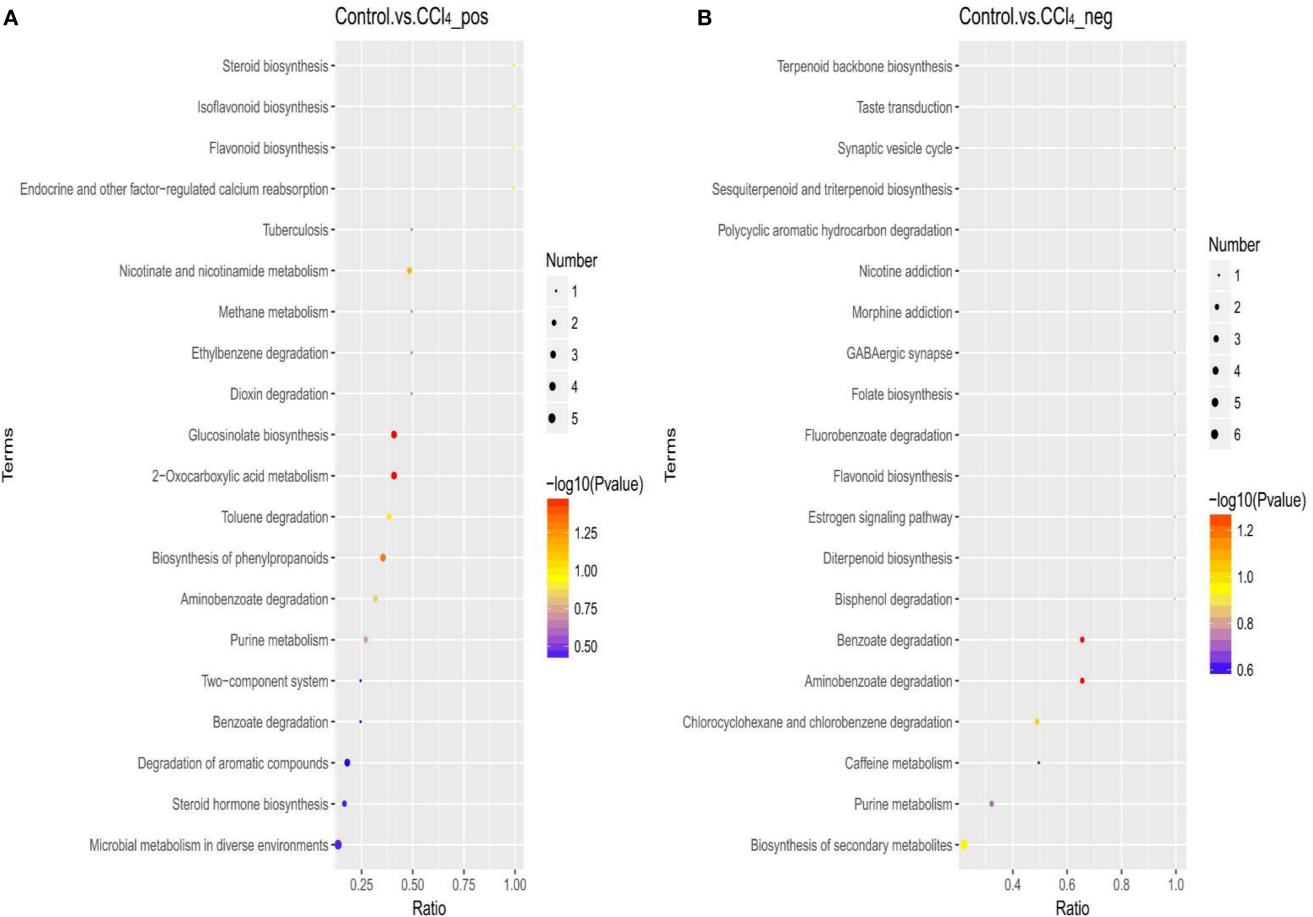
Bubble plots for identification of the most relevant metabolic pathways between the control and CCl_4_ groups under the positive **(A)** and negative **(B)** ion models.

**Figure 12:**
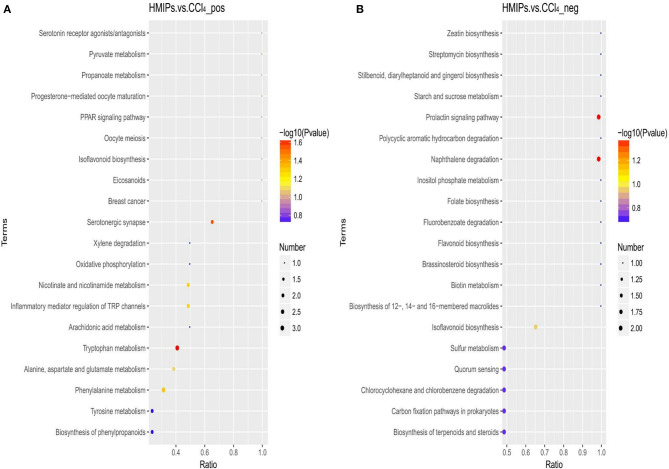
Bubble plots for identification of the most relevant metabolic pathways between the HMIP and CCl_4_ groups under the positive **(A)** and negative **(B)** ion models.

The metabolic pathway analysis (MetPA) of the candidate metabolites in Tables 2, 3 was carried out to explore the relevance of the pathways linked to the differential metabolites. The bubble map of MetPA indicated that the dramatically disturbed metabolic pathways between the control and CCl_4_ groups included flavonoid and isoflavonoid biosynthesis; steroid biosynthesis; alanine, aspartate, and glutamate metabolism; nicotinate and nicotinamide metabolism; and benzoate and aminobenzoate degradation (Figure 11). Furthermore, there were five such metabolic pathways between the HMIP and CCl_4_ groups, namely, isoflavonoid biosynthesis, tryptophan metabolism, phenylalanine metabolism, naphthalene degradation, and benzoate and aminobenzoate degradation (Figure 12).

## Discussion

Finding natural sources of hepatoprotective medicines is of keen research interest in ensuring liver protection and thereby its roles in regulating numerous physiological processes, including metabolism, synthesis, and detoxification (Bing-Ya et al., 2015; Lu et al., 2016; Yang et al., 2019). *M. importuna* mushrooms are rich sources of many bioactive components, such as polysaccharides, dietary fiber, and proteins (Gursoy et al., 2009). In particular, polysaccharides from *M. importuna* have shown multiple pharmacological and biological activities, including antioxidant, antimicrobial, antiviral, and hepatoprotective activities (Heleno et al., 2013). However, how MIPs confer protection against oxidative injury remains unknown. The animal model of acute liver injury induced by CCl_4_ is one of the classical models used to screen antioxidative medicines. Therefore, we aimed to evaluate the suitability as potent medicinal foods by examining the effects of LMIPs and HMIPs on CCl_4_-induced acute liver damage in mice.

Weight loss after CCl_4_ intoxication is considered to be caused by the direct toxicity of CCl_4_ or the indirect toxicity associated with liver damage (Yang et al., 2010). However, BW was not decreased by CCl_4_ intoxication and marginally increased by MIP administration in this study. Marked swelling of the CCl_4_-treated liver occurred, as evidenced by the enhancement of liver weight. In comparison, a significant decrease in liver weight was observed in all mice infused with HMIPs. Clinical assessment of liver injury involves measurement of the ALT, AST, TG, TC, and TBA, which in part, reflect the degree of hepatocyte damage and necrosis. This study demonstrated that the levels of these markers were elevated by CCl_4_ intoxication. MIPs inhibited the leakage of these enzymes from the liver tissue into the bloodstream. CCl_4_ is decomposed into numerous free radicals, such as trichloromethyl, after its metabolization by cytochrome P450 in hepatocytes (He et al., 2016). Due to the free radical-mediated destruction and increased permeability of the cellular membrane structure, high levels of ALT and AST enzymes infiltrate into the bloodstream, significantly promoting the activity of these enzymes in the serum. Concurrently, the trichloromethyl peroxyl radical is generated by the reaction of trichloromethyl radical with molecular oxygen and can attack liver microsomal lipid and hepatocyte membrane phospholipid molecules, leading to the initiation of the lipid peroxidation reaction (Bruinsma et al., 2015; Shi-Bin et al., 2015). High levels of TG and TC were deposited in hepatocytes, which was concomitant with an increase in the serum levels of TG and TC (Lykkesfeldt, 2007).

Lipid peroxidation resulting from oxidative stress produces a large amount of MDA, which can also contribute to hepatocyte damage, thereby aggravating the lesions (Tsikas, 2017). PC is the product of damage to amino acid side chains by oxygen-free radicals. With the aggravation of oxidative stress, the protein carbonylation becomes more severe, which impedes protein folding and promotes protein–protein cross-linking. Thus, PC is considered an effective index to evaluate the degree of protein oxidation. In contrast, SOD and CAT are important antioxidant enzymes, responsible for scavenging free radicals and inhibiting lipid peroxidation, thus protecting the hepatocytes against CCl_4_-mediated toxicity (Dmitry et al., 2017). In this study, the excessive increase in the hepatic MDA and PC contents in the CCl_4_ group were markedly attenuated by HMIP administration. Furthermore, MIPs dose-dependently alleviated the significantly decreased concentration of SOD caused by CCl_4_ intoxication. Hence, MIP administration preserved hepatic health by attenuating the oxidative damage in the liver.

In addition, the inflammatory response observed during CCl_4_-induced hepatic damage may be accompanied by the release of several cytokines (i.e., IL-1, IL-6, and TNF-α), contributing to a degenerative scenario (Sha et al., 2020). The MPO activity serves as a marker for inflammatory cell infiltration and indicates the degree of inflammation (Chen et al., 2020). Our data showed a dramatic increase in the levels of IL-1β, IL-6, TNF-α, and MPO by CCl_4_ intoxication. However, MIP administration led to a marked decrease in pro-inflammatory cytokines and MPO compared with the CCl_4_ group. Moreover, H&E staining confirmed that CCl_4_ intoxication induced acute liver damage, as indicated by vacuolation, the ballooning of hepatocytes, and inflammatory cell infiltration. HMIP administration mitigated these detrimental injuries, which further supported the aforementioned hepatoprotective effects. These results supported that MIPs possess potent hepatoprotective activity by inhibiting oxidative stress and inflammation.

Based on the more favorable inhibitory effects of HMIPs over LMIPs on CCl_4_-induced liver injury, a UHPLC–MS/MS-based metabolomic analysis was performed to evaluate the protective effect of HMIPs against CCl_4_-induced hepatic damage. The observed trends in serum biomarkers following CCl_4_ intoxication or HMIP administration are shown in Tables 1, 2. These metabolites were mainly involved in flavonoid biosynthesis, amino acid metabolism, energy metabolism, and toxicant degradation. Numerous studies have indicated multifunctional flavonoids isolated from Chinese medicines and plants as potential therapeutic agents for the improvement of the liver tissue via antioxidant, anti-inflammatory, and antifibrotic mechanisms (Gou et al., 2020; Wu et al., 2020a). Serum metabolomic analysis revealed that metabolites (i.e., isoliquiritigenin and prunin) related to flavonoid biosynthesis were significantly reduced by CCl_4_ intoxication. However, the ratio of prunin between the HMIP and CCl_4_ groups was lower than 1.0, perhaps indicating that HMIP administration did not affect flavonoid biosynthesis.

The liver is a vital organ in regulating amino acid metabolism, and any dysfunction in the liver could cause a disturbance in the amino acid concentrations (Waters et al., 2005). The decreased ratio of γ-aminobutyric acid observed between the control and CCl_4_ groups in this study indicated that CCl_4_ infusion might disturb the alanine, aspartate, and glutamate metabolism because γ-aminobutyric is one of the metabolites in this pathway. Tryptophan is metabolized along the kynurenine and serotonin pathways, while phenylacetylglycine participates in phenylalanine metabolism (Kamiguchi et al., 2017; Li et al., 2017). In this study, amino acids in the serum were promoted by CCl_4_ intoxication, perhaps because of decreased protein synthesis and increased protein catabolism (Guo et al., 2017). CCl_4_ intoxication can disturb methylation pathways such as the hypomethylation of ribosomal RNA, leading to defective protein synthesis (Clawson et al., 1987). Moreover, protein catabolism plays an important role in regulating lipid and glucose metabolism, which could induce cytokine secretion under abnormal conditions (Sinha et al., 2020).

Trigonelline (*N*-methylnicotinic acid) and 6-hydroxynicotinic acid are intermediates of nicotinate and nicotinamide metabolism, respectively. Nicotinamide has been used as a metabolic precursor of nicotinamide adenine dinucleotide (NAD^+^) biosynthesis to raise the intracellular NAD^+^ pool for improvement of metabolic diseases involving glycolysis (French, 2015; Wan et al., 2019). Trigonelline showed a hepatoprotective effect against non-alcoholic fatty liver diseases by reducing oxidative stress and pro-apoptosis (Zhang et al., 2015). In this study, we discovered that trigonelline was impeded by CCl_4_ intoxication as evidenced by the ratio between the control and CCl_4_ groups (Table 1). However, the reduced levels of trigonelline could not be reversed by HMIP infusion. Additionally, it was reported that some commonly used medications, such as *p*-aminobenzoate, an antifibrotic agent, may unpredictably be the underlying cause of liver injury (Al Attar and Kilgore, 2018). In our study, catechol, a strong toxicant with carcinogenic properties, was decreased by HMIP administration. Thus, we speculated that HMIPs might beneficially facilitate toxicant degradation to protect the liver tissues. In brief, HMIPs showed hepatoprotective activity against liver injury by affecting amino acid metabolism, energy metabolism, and toxicant degradation. However, the underlying mechanisms for these findings remain unclear and require elucidation.

## Conclusion

Taken together, our findings demonstrated that MIP administration alleviated CCl_4_-induced hepatic injury by promoting antioxidant and anti-inflammatory activities. Furthermore, the metabolomic analysis showed that HMIPs regulate the pathways of amino acid metabolism, energy metabolism, and toxicant degradation to subvert the disturbances induced by CCl_4_. This study provides a scientific basis for MIPs as a curative treatment for hepatic injury.

## Data Availability Statement

The raw data supporting the conclusions of this article will be made available by the authors, without undue reservation.

## Ethics Statement

The animal study was reviewed and approved by the Ethics Review Committee for Animal Experimentation of Sichuan Academy of Agricultural Sciences.

## Author Contributions

YX, BG, and WP conceived and designed the experiments. YX, LX, JT, and YC conducted the experiments. YX, XH, ZZ, and JZ analyzed the data. YX and WP wrote the article. All authors have read and agreed to the published version of the manuscript.

## Conflict of Interest

The authors declare that the research was conducted in the absence of any commercial or financial relationships that could be construed as a potential conflict of interest.

## Publisher's Note

All claims expressed in this article are solely those of the authors and do not necessarily represent those of their affiliated organizations, or those of the publisher, the editors and the reviewers. Any product that may be evaluated in this article, or claim that may be made by its manufacturer, is not guaranteed or endorsed by the publisher.
